# Dynamic connectivity states estimated from resting fMRI Identify differences among Schizophrenia, bipolar disorder, and healthy control subjects

**DOI:** 10.3389/fnhum.2014.00897

**Published:** 2014-11-07

**Authors:** Barnaly Rashid, Eswar Damaraju, Godfrey D. Pearlson, Vince D. Calhoun

**Affiliations:** ^1^The Mind Research Network, AlbuquerqueNM, USA; ^2^Department of Electrical and Computer Engineering, University of New MexicoAlbuquerque, NM, USA; ^3^Olin Neuropsychiatry Research Center – Institute of Living, HartfordCT, USA; ^4^Departments of Psychiatry, Yale University School of MedicineNew Haven, CT, USA; ^5^Departments of Neurobiology, Yale University School of MedicineNew Haven, CT, USA

**Keywords:** dynamic functional connectivity, intrinsic connectivity networks, independent component analysis, schizophrenia, bipolar disorder

## Abstract

Schizophrenia (SZ) and bipolar disorder (BP) share significant overlap in clinical symptoms, brain characteristics, and risk genes, and both are associated with dysconnectivity among large-scale brain networks. Resting state functional magnetic resonance imaging (rsfMRI) data facilitates studying macroscopic connectivity among distant brain regions. Standard approaches to identifying such connectivity include seed-based correlation and data-driven clustering methods such as independent component analysis (ICA) but typically focus on average connectivity. In this study, we utilize ICA on rsfMRI data to obtain intrinsic connectivity networks (ICNs) in cohorts of healthy controls (HCs) and age matched SZ and BP patients. Subsequently, we investigated difference in functional network connectivity, defined as pairwise correlations among the timecourses of ICNs, between HCs and patients. We quantified differences in both static (average) and dynamic (windowed) connectivity during the entire scan duration. Disease-specific differences were identified in connectivity within different dynamic states. Notably, results suggest that patients make fewer transitions to some states (states 1, 2, and 4) compared to HCs, with most such differences confined to a single state. SZ patients showed more differences from healthy subjects than did bipolars, including both hyper and hypo connectivity in one common connectivity state (dynamic state 3). Also group differences between SZ and bipolar patients were identified in patterns (states) of connectivity involving the frontal (dynamic state 1) and frontal-parietal regions (dynamic state 3). Our results provide new information about these illnesses and strongly suggest that state-based analyses are critical to avoid averaging together important factors that can help distinguish these clinical groups.

## INTRODUCTION

Schizophrenia (SZ) and bipolar disorder (BP) are two common psychiatric conditions characterized by gray and white matter abnormalities and disrupted connectivity across large-scale brain networks (Mohamed et al., 1999; Kubicki et al., 2007). Such dysconnectivity includes disruption of both structural (Kubicki et al., 2007; Rotarska-Jagiela et al., 2008, 2009) and functional connectivity (FC; Meyer-Lindenberg et al., 2001; Uhlhaas and Singer, 2006; Garrity et al., 2007; Calhoun et al., 2008a, 2011) that may be related to clinical symptoms, including cognitive dysfunction. SZ is often referred to as a dysconnection syndrome, where the term “dysconnection” refers to over- or under-connection of neural circuits with respect to a healthy control group (Friston et al., 1993). Because changes in the function of a single brain region cannot explain the range of impairments observed in SZ or BP (Achim and Lepage, 2005; Van Snellenberg et al., 2006; Minzenberg et al., 2009; Ragland et al., 2009; Wang et al., 2009; Chepenik et al., 2010), researchers need to identify altered connectivity in relevant core brain networks.

Recently, FC has been used to examine the functional organization of brain networks in various psychiatric illnesses, where FC is defined as the temporal covariance of neural signals between multiple spatially distinct brain regions (Friston et al., 1993). Different analytic tools have been applied to resting-state fMRI data to describe brain FC, including seed-based analysis (Biswal et al., 1995; Greicius et al., 2003), data-driven methods, such as independent component analysis (ICA; Hyvärinen and Oja, 2000; Calhoun et al., 2001b, 2009; Damoiseaux et al., 2006; Fox and Raichle, 2007; Calhoun and Adali, 2012), clustering (Cordes et al., 2002), multivariate pattern analysis (MVPA; Norman et al., 2006; Zhu et al., 2008; Zeng et al., 2012), graph theory (Achard et al., 2006; Buckner et al., 2009) and centrality (Lohmann et al., 2010). In seed-based approach, the connectivity patterns are based on a selected seed region of interest (ROI), while ICA-based methods do not require prior knowledge of brain activity or seed ROI selection (Erhardt et al., 2011a).

Until recently, most fMRI studies assumed that FC is stationary throughout the entire scan period (or at least static during a giving task or condition such as rest). This assumption of stationarity is likely an oversimplification since it is likely that individuals are engaged in slightly different mental activities at different points in time. In addition, previous works showing evidence of fluctuation in FC (Arieli et al., 1996; Makeig et al., 2004; Onton and Makeig, 2006) are consistent with the idea that dynamic changes in FC occur during the course of the experiment. Recent studies show that connectivity dynamics can capture uncontrolled but reoccurring patterns of interactions among intrinsic networks during a task or at rest (Sakoǧlu et al., 2010; Allen et al., 2012; Hutchison et al., 2013; Calhoun et al., 2014). These studies provide results that cannot be detected with static FC analyses. In a dynamic connectivity study using wavelet-based time-frequency coherence analysis, significant results were observed for resting state connectivity variation between posterior cingulate cortex and an anti-correlated network (Chang and Glover, 2010). Another approach for studying dynamic connectivity is the sliding-window correlation technique (Allen et al., 2012; Hutchison et al., 2013).

Resting state BOLD studies have proven useful recently to investigate abnormal FC, as the absence of a specific task complements task-specific study by measuring intrinsic functional brain organization without any differential behavioral performance and task activity between diagnostic groups, and thus makes it easier for cognitively compromised patients to participate in such studies. Resting-state fMRI connectivity has been used to identify differences in multiple patient groups including SZ (Calhoun et al., 2009, 2011; Sakoǧlu et al., 2010; Damaraju et al., 2014), BP (Calhoun et al., 2011), Alzheimer’s disease (Greicius et al., 2004; Sorg et al., 2007), autism (Starck et al., 2013), and others. However, to our knowledge, no study to date has evaluated changes in connectivity patterns over time in fMRI in patient groups versus controls. It is not yet known how spatial and temporal dynamics of resting state networks contribute to individual psychopathological disorders. Both SZ and BP are diagnosed using cross-sectional clinical symptoms along with longitudinal course and outcome measures. There are significant overlaps in symptoms and disease progression between these two disorders that can make it difficult to differentiate them without repeated clinical diagnostic assessment (Keshavan et al., 2011). By determining a reliable diagnostic indicator (‘biomarker’) based on biological features of these diseases, a baseline for developing more accurate and reliable differentiating tools for diagnosis, and ultimately treatment, can in theory be provided (Keshavan et al., 2013).

Previous studies show both similarities and differences in static FC between SZ and BP. Most prior studies focused on quantifying the underlying characteristics of sensory, auditory, cognitive control (CC) and emotional processes of the brain. For example, the default mode network (DMN), consists of a set of brain regions known to be activated during internally focused tasks and may be involved in processes such as attention to internal emotional states, self-referential processing or task- independent thoughts (Buckner et al., 2008). DMN data may distinguish between SZ and BP (Öngür et al., 2010; Calhoun et al., 2011). There are numerous studies suggesting abnormal default network connectivity in SZ and BP (Zhou et al., 2007, 2008; Calhoun et al., 2008b, 2011), although both increased and decreased connectivity have been reported. Different analytical techniques could account for these inconsistent findings, as seed-based and data-driven analyses and varying preprocessing steps do not necessarily produce the same results. Also each intrinsic brain network comprises a collection of multiple network components, only a few of which might be affected throughout a specific period of illness.

Prior studies have identified abnormal connectivity in other intrinsic networks. For example, patients with persistent auditory verbal hallucinations may have increased connectivity in the cingulate cortex within the speech-related network (Wolf et al., 2011). In attention and executive control networks, patients demonstrated abnormal connectivity in precuneus and right lateral pre-frontal areas. Few studies have examined both bipolar and SZ patients. A recent study of both disorders (Öngür et al., 2010; Meda et al., 2012; Khadka et al., 2013) found subgenual and medial prefrontal anomalies in BP patients and dorsal medial prefrontal anomalies in SZ patients, although considerabe overlap among groups.

In this paper, we implement a recently published approach to assess functional network connectivity (FNC) dynamics between healthy controls (HCs) and SZ and bipolar patients, which includes group spatial ICA, dynamic FNC via sliding time window correlation, and k-means clustering of windowed correlation matrices (Allen et al., 2012). We hypothesized that disrupted functional integration in SZ and bipolar patients can be found in several brain regions including temporal, frontal, visual, and DMNs as suggested by previous studies. To test our hypothesis we conducted group difference analyses in connectivity using independent two sample *t*-tests. The results show that dynamic FNC captured by sliding time window analysis can reveal significant differences between patients and controls that cannot be found using conventional stationary FNC analysis.

## MATERIALS AND METHODS

### PARTICIPANTS

We assessed 159 total subjects comprising 61 screened HCs [HC, age 35.44 ± 11.57 (range), 28 females], 60 patients diagnosed with SZ or schizoaffective disorder (SZ, age 35.85 ± 12.01, 13 females) and 38 bipolar subjects (BP, age 38.96 ± 10.90, 20 females), matched for age with no significant differences among three groups, where age: *p* = 0.303, *F* = 1.2031, DF = 2. Significant differences in sex among three groups were found, where sex: *p* = 0.002, *X*^2^ = 11.81, DF = 2. Diagnoses were based on detailed medical and psychiatric history, chart reviews, and the Structured Clinical Interview for DSM Disorders (Gibbon et al., 1997). None were acutely ill at the time of scanning. Bipolar patients were a mixture of psychotic and non-psychotic by history.

#### Data acquisition

Resting-state fMRI scans were acquired at the Institute of Living, Hartford, CT, USA on a 3T Siemens Allegra head-only scanner with 40 mT/m gradients and a quadrature head coil. T2* -weighted functional images were acquired using gradient echo planar imaging (EPI) method with repetition time (TR) = 1.5 s, echo time (TE) = 27 ms, field of view = 24 cm, acquisition matrix 64 × 64, flip angle = 700, voxel size = 3.75 mm × 3.75 mm × 4 mm, slice thickness = 4 mm, gap = 1 mm, number of slices = 29, 210 frames and ascending acquisition. Subjects were instructed to keep their eyes open, look at a fixation cross on a monitor display and to rest quietly during the scan session.

### DATA PRE-PROCESSING

Functional images were pre-processed using an automated pipeline based around SPM 5^1^. Pre-processing included the removal of the first four image volumes to avoid T1 equilibration effects, realignment using INRIalign (Freire et al., 2002), slice-timing correction using the middle slice as the reference frame, spatial normalization into Montreal Neurological Institute (MNI) space^2^, reslicing to 3 mm × 3 mm × 3 mm voxels, and smoothing with a Gaussian kernel (FWHM = 5 mm). Voxel timeseries were z-scored to normalize variance across space, minimizing possible bias in subsequent variance-based data reduction steps (Allen et al., 2012).

In order to limit the impact of motion we excluded from analysis subject data with a maximum translation of >2 mm or with SFNR <275 (Signal-to-fluctuation-noise ratio, where the signal is the average voxel intensity in all the ROIs defined in the object, averaged across time, and the fluctuation noise is the temporal standard deviation of the spatial mean in the same ROIs, after removing the slow drift from the temporal series). Patient and control groups were age matched. Additional processing steps were taken to mitigate against residual motion effects as described later.

### GROUP ICA AND POST-PROCESSING

Imaging data were decomposed into functional networks using a group-level spatial ICA (Calhoun et al., 2001a; Calhoun and Adali, 2012). Group ICA was performed using the GIFT toolbox (Calhoun, 2004). In order to obtain functional parcellation, we used a high model order ICA (number of components, *C* = 100) to decompose the functionally homogeneous cortical and subcortical regions exhibiting temporally coherent activity (Kiviniemi et al., 2009; Smith et al., 2009; Abou-Elseoud et al., 2010). In the subject-specific data reduction principle component analysis (PCA) step, 120 principal components were retained (retaining >99% of the variance of the data). Group data reduction retained *C* = 100 PCs using the expectation-maximization (EM) algorithm as implemented in the GIFT toolbox. The Infomax group ICA (Calhoun et al., 2001b) algorithm was repeated 20 times in ICASSO (Himberg and Hyvarinen, 2003) and the resulting components were clustered to estimate the reliability of the decomposition (Himberg et al., 2004). Subject-specific spatial maps (SMs) and time-courses (TCs) were estimated using the GICA1 back-reconstruction method based on PCA compression and projection (Calhoun et al., 2001b; Erhardt et al., 2011b). Out of the 100 components obtained, we characterized 49 components as ICNs that depicted peak cluster locations in gray matter with minimal overlap with white matter, ventricles and edges of the brain and also exhibit higher low frequency temporal activity. Subject specific time courses and spatial maps were obtained via back reconstruction.

Additional post-processing steps including linear, quadratic and cubic detrending, multiple regression of the six realignment parameters and their temporal derivatives, removal of detected outliers, and low-pass filtering with a high frequency cutoff of 0.15 Hz were applied to the component TCs in order to remove trends associated with scanner drift and movement-related artifacts. We have detected the outliers based on the median absolute deviation, as implemented in 3D DESPIKE (Cox, 1996). Outliers were replaced with the best estimate using a third-order spline fit to the clean portions of the TCs.

### FC ESTIMATION

**FIGURE 1:**
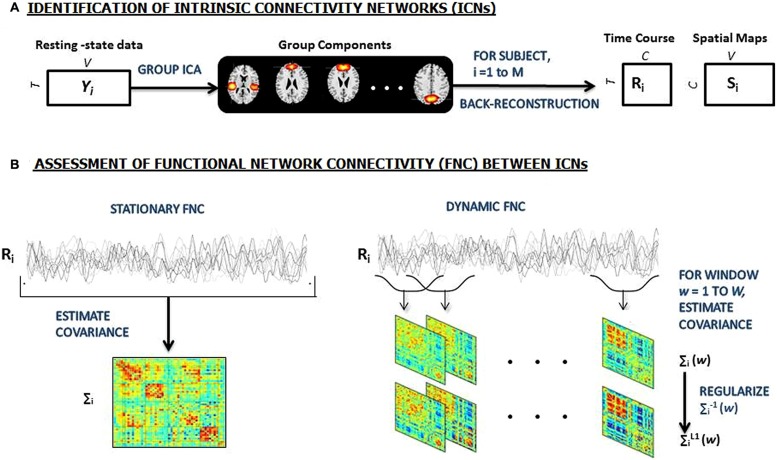
**(A)** An overview of the sliding window analysis. Group independent component analysis (ICA) is used to decomposed resting-state data from 159 subjects into 100 components, 49 of which are identified as intrinsic connectivity networks (ICNs). GICA1 back-reconstruction method is used to estimate the subject specific spatial maps (SMs) and time courses (TCs). **(B)** Stationary FC between components (left) is estimated as the covariance of TCs. Dynamic FC (right) is estimated as the series of regularized covariance matrices from windowed portions of each subject’s component TCs and then the matrices are aggregated across subjects [Adapted from (Allen et al., 2012)].

The static FNC for each subject was estimated from the TC matrix, as the C × C sample covariance matrix (see **Figure 1A**). In addition to the standard FNC analyses, we computed correlations between ICN TCs using a sliding temporal window [Tukey window (see **Figure 1B**)] having a width of 22 TRs = 33 s; sliding in steps of 1 TR), resulting in *W* = 180 windows to capture the variability in connectivity. To characterize the full covariance matrix, we estimated covariance from the regularized precision matrix or the inverse covariance matrix (Smith et al., 2011). Following the graphical LASSO method of (Friedman et al., 2008), we placed a penalty on the L1 norm of the precision matrix to promote sparsity. The regularization parameter lambda was optimized separately for each subject by evaluating the log-likelihood of unseen data (windowed covariance matrices from the same subject) in a cross-validation framework. Final dynamic FC estimates for each window, were concatenated to form a C × C × W array representing the changes in covariance (correlation) between components as a function of time.

### DYNAMIC STATES AND CLUSTERING

From all of the dynamic windowed FNC matrices, we selected windows of higher variability as subject exemplars and used K-means clustering to obtain group centrotypes. We repeated the clustering method using different distance functions (correlation, cosine, rather than the L1-norm) and also found very similar results. We determined the number of clusters to be five using the elbow criterion of the cluster validity index, which is computed as the ratio between within-cluster distances to between-cluster distance. These centrotypes are then used as starting points to cluster all of the dynamic FNC data. Group specific centrotypes were computed. Subject specific centrotypes were used to perform independent sample *t*-tests to probe for group differences.

## RESULTS

### INTRINSIC CONNECTIVITY NETWORKS (ICNs)

**FIGURE 2:**
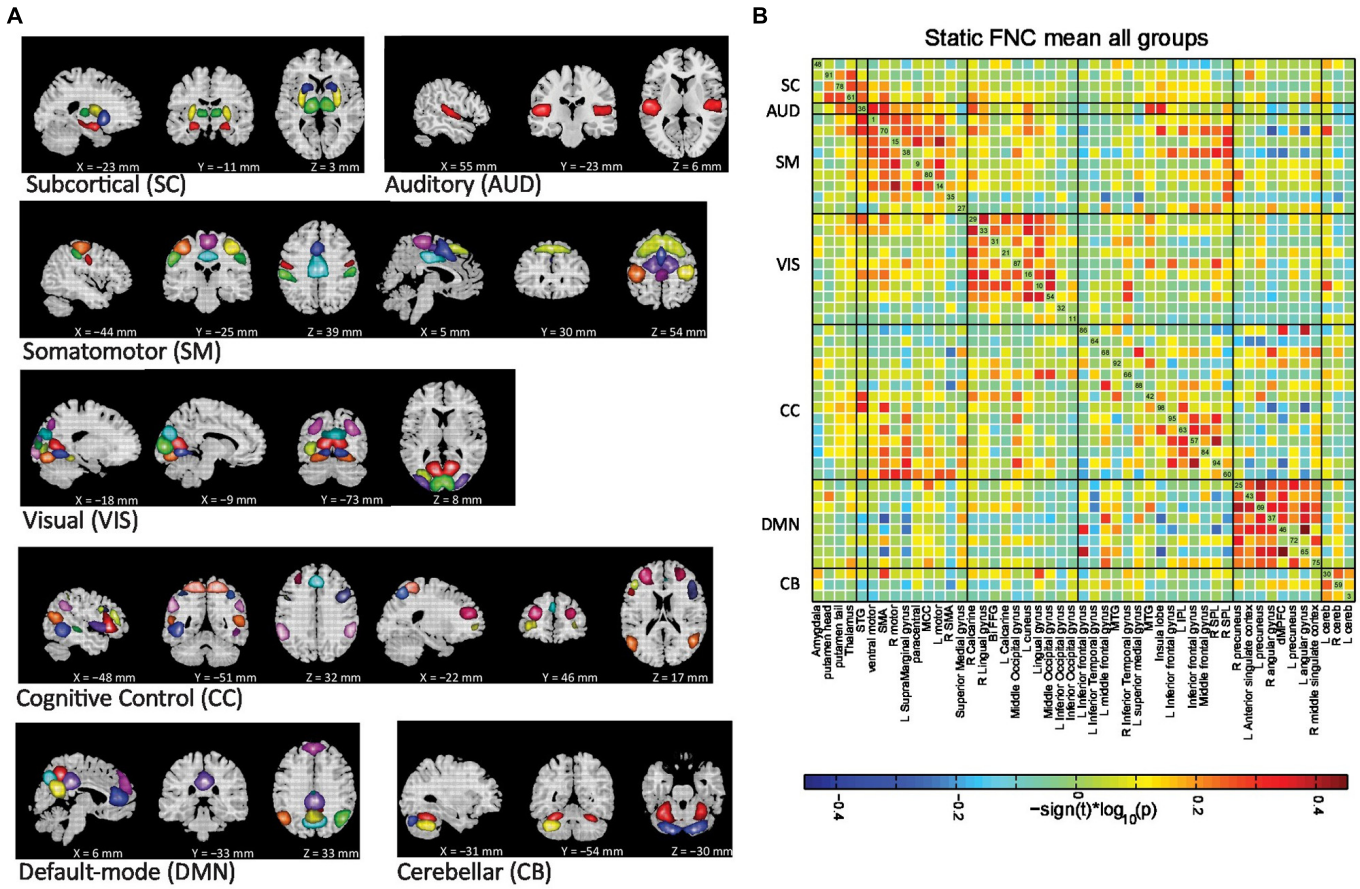
**(A)** Non-artifactual ICNs and **(B)** Group mean static FNC between ICN timecourses. ICNs are divided into groups and arranged based on their anatomical and functional properties. FC was averaged over all subjects and displayed as inverse Fisher-transformed. All of the ICN labels in **(B)** indicate the brain region with peak amplitude and should be considered as bilateral activation unless mentioned as left (L) or right (R). See table S1 for more detailed information on each intrinsic component. STG, superior temporal gyrus; SMA, supplementary motor area; MCC, middle cingulated cortex; Bi-FFG, bi-fusiform gyrus; MTG, middle temporal gyrus; IPL, inferior parietal lobule; SPL, superior parietal lobule; dMPFC, dorso-medial prefrontal cortex; Cereb, cerebellum.

ICA was successfully used to identify the intrinsic connectivity networks (ICNs) in HCs and patients with SZ and bipolar, and to identify differences in FNC among these ICNs. The spatial maps of 49 ICNs identified with group ICA are shown in **Figure 2A**. ICNs are grouped by their anatomical and functional properties, which include the following: sub-cortical (SC), auditory (AUD), sensorimotor (SM), visual (VIS), CC, default mode (DM), and cerebellar (CB) components. The observed ICN networks are very similar to those found in previous studies with low model order ICA (Calhoun et al., 2008a) as well as high model order ICA (Kiviniemi et al., 2009; Smith et al., 2009; Allen et al., 2011).

The SC networks are represented by four components (ICs 48, 91, 78, and 61) with activations focused in the amygdala, putamen head, putamen tail, and thalamus. The AUD network is represented by a single component (IC 36) with bilateral activation of the superior temporal gyrus (STG). The SM regions are captured by nine components (ICs 1, 70, 15, 38, 9, 80, 14, 35, and 27). The visual system (VIS) is represented by ten components (ICs 31, 10, 11, 16, 21, 29, 32, 33, 54, and 87), which matches with the functional and structural characterization of occipital cortex. The cognitive control network (CC) includes the ICN components involved in directing and monitoring behavior, mediating memory and language functions (ICs 64, 66, 92, 42, 60, 63, 94, and 95). The DMN is captured by eight components. Finally, we classify the CB network with three components with activations in both right and left cerebellum.

### STATIC FNC

Group mean FC or static FNC between ICN timecourses is shown in **Figure 2B**. The ICN components in the static FNC matrix were initially ordered using algorithms in the brain-connectivity toolbox (Rubinov and Sporns, 2010) that maximize modularity of the connectivity matrix. These were manually partitioned into subgroups as in our earlier work (Allen et al., 2012). The average connectivity matrix demonstrates strong positive connectivity within subcortical, VIS, SM, default-mode, and CB networks. A set of CC regions also shows this positive connectivity among themselves and are also connected to certain VIS networks. These CC and VIS regions show anti correlation to default-mode regions. Two sample *t*-tests did not reveal any group differences in static or overall connectivity. Previous studies have found differences in FNC in similar groups, but not with such a high model order that produces more focus brain regions, but also more comparisons. In our case, several FNC pairs showed a trend level of significance, but did not quite reach a corrected level of significance for the static FNC analysis.

However, we also computed an analysis of FNC differences within groups of components (e.g., DMN components re-combined), called a network group (NG). To do this we computed, for each NG, the average connectivity between it and all other NGs (Repovs et al., 2011). We then applied an FDR correction for multiple comparisons of the between-NG connectivity. Several between-NG pairs showed significant group SZ/control differences, including sub-cortical and sensory-motor, sub-cortical and CC, and DM and cerebellum. One pair, sub-cortical and CC, showed a significant difference between SZ and BP patients. No between-NG connectivity difference was found between HCs and bipolars.

### DYNAMIC CONNECTIVITY STATES AND GROUP DIFFERENCES

**FIGURE 3:**
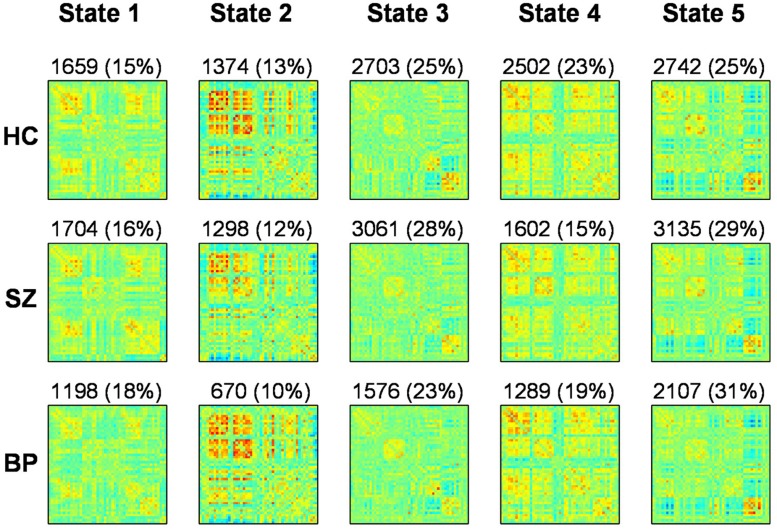
**Results from clustering approach for *k* = 5.** Group specific centroids of the states (state 1 to state 5) are obtained from k-means clustering. The total number and percentage of occurrences is listed above each centroid

We use k-means clustering method to identify re-occurring pattern of FC states (**Figure 3**). Dynamic FNC analysis suggests that patients make fewer transitions to some states (States 1, 2, and 4) compared to HCs. Significant differences were found between groups in dynamic FNC states 1, 2, 3, and 4, between healthy control and patient groups as well as between SZ and bipolar patients.

**FIGURE 4:**
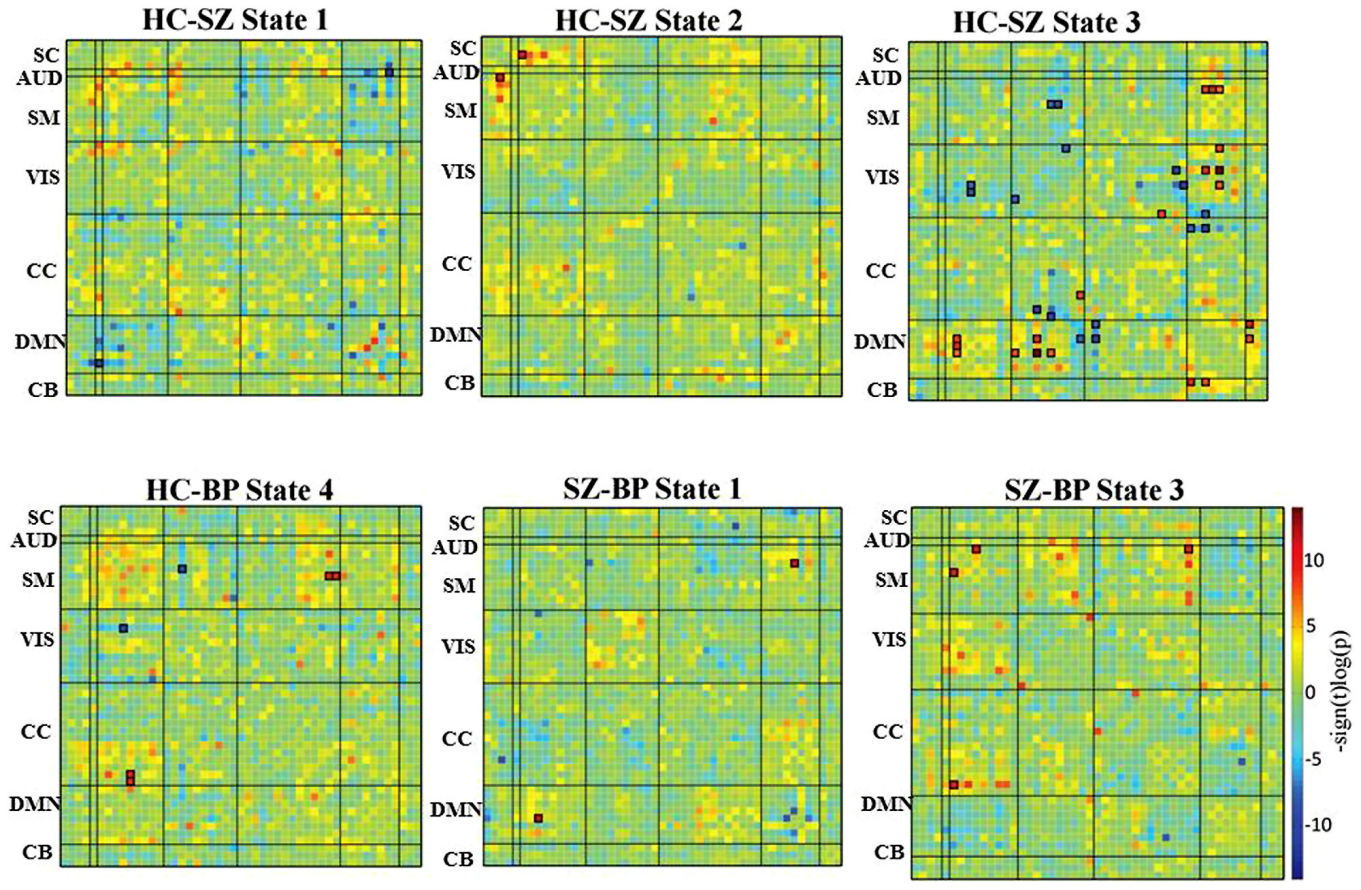
**Difference in dynamic states.** Group differences in dynamic FC states are obtained using an independent two-sample *t*-test between healthy control and patient groups, as well as between schizophrenia and bipolar patient groups. The cells that have survived a FDR threshold for multiple comparison correction are enclosed in black patch.

**FIGURE 5:**
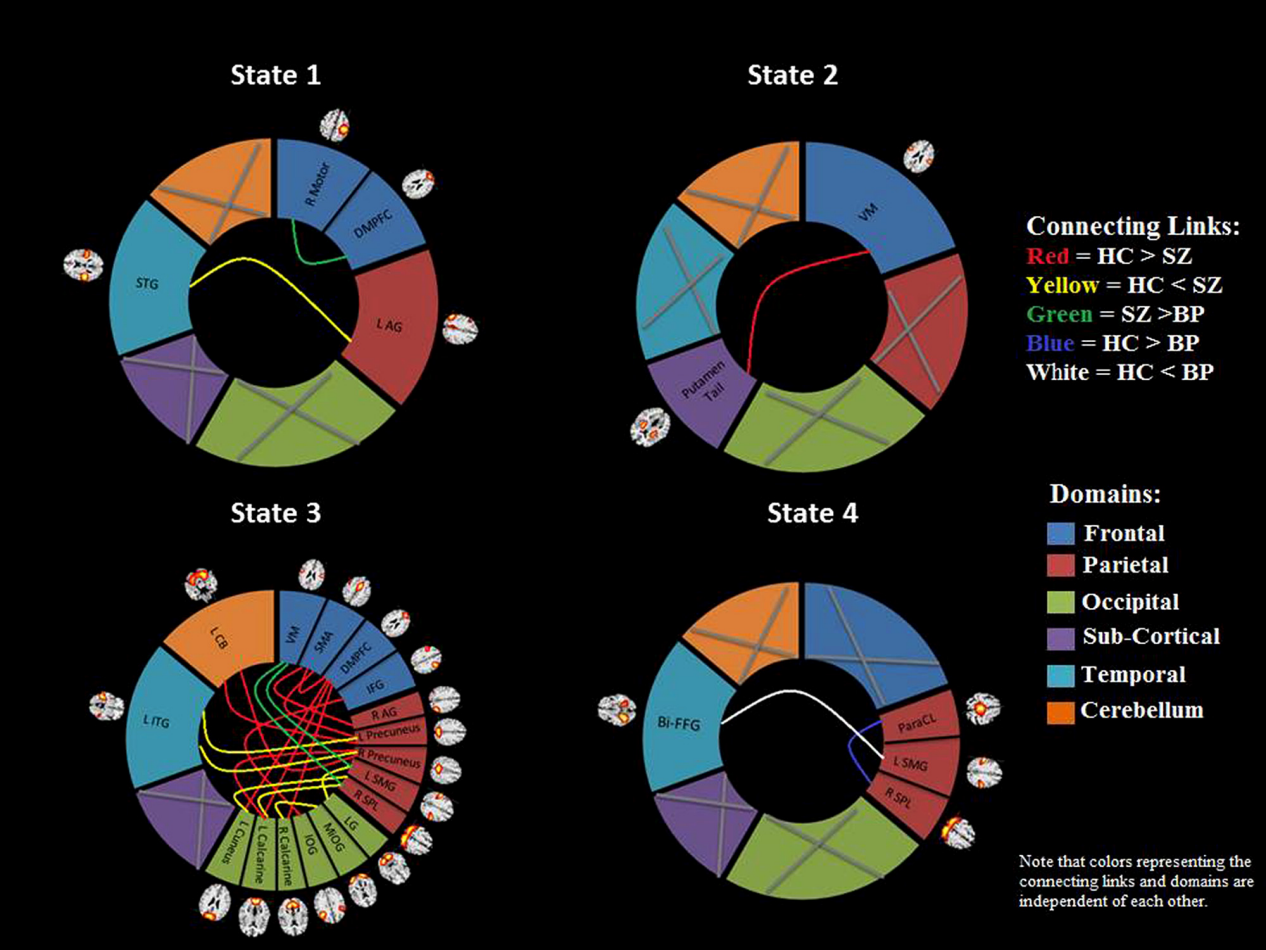
**Brain connectome.** A visual summary of significant connectivity differences in dynamic states between different ICN components for control and patient groups. The gray cross mark indicates that no component from that region showed any significant group difference. Note that the colors of the connecting links and the domains are independent of each other. All of the component labels indicate the brain region with peak amplitude and should be considered as bilateral activation unless mentioned as left (L) or right (R). STG, superior temporal gyrus; dMPFC, dorso-medial prefrontal cortex; AG, angular gyrus; VM, ventral motor; ITG, inferior temporal gyrus; CB, cerebellum; SMA, supplementary motor area; IFG, inferior frontal gyrus; SMG, supramarginal gyrus; SPL, superior parietal lobule; LG, lingual gyrus; MOG, middle occipital gyrus; IOG, inferior occipital gyrus; Bi-FFG, bi-fusiform gyrus; ParaCL, paracentral.

**FIGURE 6:**
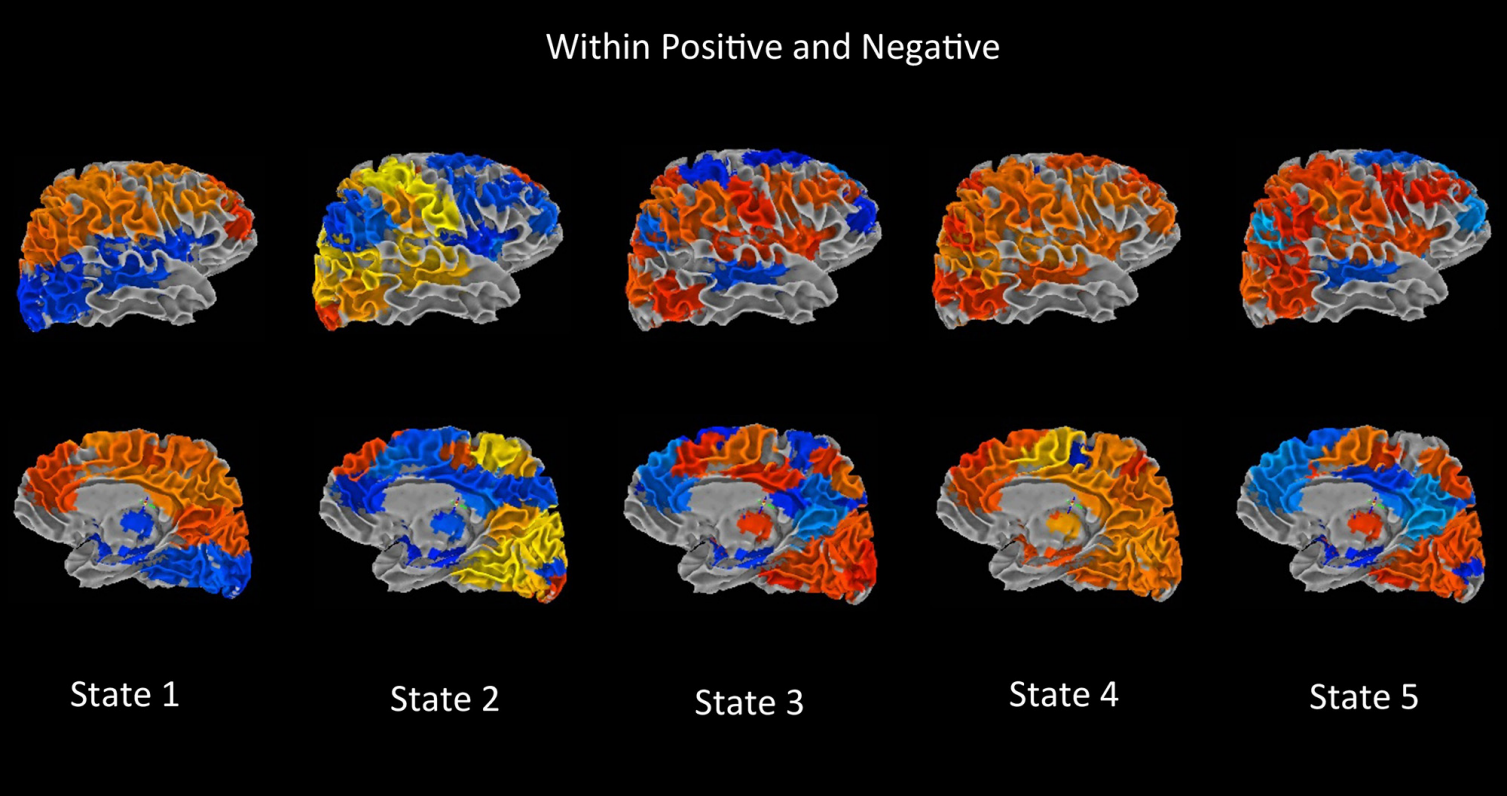
**Main dynamic effects rendering maps.** Using the Brain Connectivity Toolbox, modularity in the dynamic FC matrices was found. The FC matrix is divided into two modules with nodes of each module correlated positively with each other in general and anticorrelated with nodes of other module. For each component the average positive connectivity within a module was computed and stored as “component weight.” These weights were then used to create weighted spatial map containing all associated components for a given dynamic state, and finally the weighted spatial maps were projected onto a 3-dimensional MNI surface using the AFNI-SUMA (Saad et al., 2004).

**Figure 4** summarizes the difference between groups measured by the connectivity between ICN component pairs. For better visualization purpose, brain connectome for each of the significant dynamic states is shown in **Figure 5**. Also, **Figure 6** shows the rendering maps for main effects of dynamic connectivity for all the subjects. To create the rendering maps, we first identified the modularity in the dynamic FC matrix for each state using the Brain Connectivity Toolbox (Rubinov and Sporns, 2010). For each component, the average connectivity within a module was computed and stored as “component weight vector.” These positive or negative weights were then used to create weighted spatial map containing all contributing components for a given dynamic state, and finally the weighted spatial maps were projected onto a 3-dimensional MNI surface using the AFNI-SUMA (Saad et al., 2004).

In state 1, two component pairs captured the differences between HCs and SZ patients as well as between the two patient groups (**Figures 4** and **5**). Compared to HCs, SZ patients showed greater connectivity between the component pair STG (C36) and left angular gyrus (C65), in the temporal-parietal region. Also compared to BP, SZ patients showed greater connectivity between two frontal components: right motor (C15) and dorso-medial prefrontal cortex (DMPFC; C46).

In dynamic connectivity state 2 (**Figures 4** and **5**), HCs showed greater connectivity between a sub-cortical component, putamen tail (C78) and a frontal component, ventral motor (VM C1), compared to the patients with SZ.

In dynamic state 3 (**Figures 4** and **5**), most of the differences in connectivity were captured between HCs and SZ patients, and between the two patient groups. These connectivity differences were found in frontal, parietal, occipital, temporal and CB regions of the brain. SZ patients showed greater connectivity between several temporal-parietal components, compared to HCs. The connectivity between most of the frontal-parietal and frontal-occipital components was greater in HCs compared to SZ patients, whereas the connectivity between most of the parietal-occipital components was greater in SZ patients. Also greater connectivity in SZ was found between two frontal-parietal component pairs, VM, and left supramarginal gyrus, and VM and right superior parietal lobule (SPL), compared to BP. This is the only dynamic state that captured differences between these two patient groups.

Dynamic state 4 revealed differences between HCs and bipolar patients in temporal and parietal regions, where greater connectivity in HCs was found between two parietal components, paracentral and SPL, and greater connectivity in BP was found between a temporal component bilateral fusiform gyrus and a parietal component left supramarginal gyrus. Dynamic state 5 did not display any significant group differences in FC. Also, no significant correlation between symptoms and connectivity was found.

## DISCUSSION

We explored dynamic FC patterns with ICA, sliding windows, and clustering. Our analysis of connectivity dynamics in a relatively large sample (*n* = 159) provides, to our knowledge, the first whole-brain characterization of regional differences in FC variability and distinction of discrete FC states among healthy control, SZ, and bipolar patients. We identified several ICNs that differentiate SZ and BP from HCs.

Dynamic FC captures stable connectivity patterns that are not observed in the stationary FC. FC of the brain is not stationary; rather it’s changing over time. Thus observing group-wise differences in connectivity across time as captured by the discrete dynamic states gives us more valuable information that cannot be found within the stationary or mean FC.

In **Figure 3**, each matrix represents the centroid of a cluster and signifies a connectivity state stably present within data. These dynamic connectivity states are fully reproducible and present in numerous subjects. Dynamic state 1 resembles the pattern of stationary FC. FC patterns in state 2–5 represent connectivity show considerable deviation from the mean FC.

One of the notable features that differ between FC states is the connectivity within DMN regions and, between DMN and other functional networks. In state 3 and 5, the DMN regions show strong synchronous activation with themselves, and mostly asynchronous activation with other functional networks. Particularly in state 3, the DMN regions show strong asynchrony with most of the CC components. State 5 shows the similar nature of connectivity between DMN and CC components, but with a reduced number of CC components. Also in states 3 and 5, several sensori-motor components show negative correlations with the DMN system, which is not visible in other states. In contrast, state 1, 2, and 4 do not show similar FC patterns between DMN and other ICN networks, where segregation of synchronized activation between DMN and other ICN nodes can be observed.

State 2 captures the FC differences between cortical and subcortical components, where strong asynchronous activation between subcortical regions (amygdala, putamen head, putamen tail, and thalamus) and sensori-motor, auditory, and VIS cortex were found. Cerebellum also shows this asynchrony with these cortical regions. Also substantial reduction in connectivity between DMN regions can be observed in this state. As mentioned in several previous studies, reduced thalamocortical connectivity (Spoormaker et al., 2010), increased subcortical connectivity (Larson-Prior et al., 2011) and a segregation of DMN connectivity (Spoormaker et al., 2010; Larson-Prior et al., 2011) indicated a state of light sleep or drowsiness. Also similar dynamic state related to drowsiness was found among healthy subjects in (Allen et al., 2012).

Hutchison et al. (2013), periods of hypersynchronization were described where extremely high intra-network connectivity between all nodes of oculomotor and motor networks were found in macaques and humans. This relates well to our observed discrete FC states where states 1,2, and 4 show time windows with high correlations throughout the motor system (and some motor components in state 5), while state 3 and 5 represent periods with synchronous activation between VIS areas. From our results, we can predict that periods of hypersynchronization between motor nodes would also include synchronous activation of DM regions and segregated synchronous activation between the nodes in other ICNs. Also, we can predict that hypersynchronization between VIS areas will be accompanied by synchronization of DMN regions and strong asynchronous activation with other functional networks.

Note that, state 4 is the only dynamic state where we have found significant differences between healthy control and bipolar subjects. State 4 shows synchronous activity within most of the network nodes except few VIS and CC components, which show anti-correlation with themselves as well as with other ICN networks. The differences between HC and BP were captured between a pair of SM component (paracentral gyrus) and CC component(R SPL), and between a pair of SM component (left supplementary motor area) and VIS component (bi-fusiform gyrus).

The differences between groups are not localized in a single dynamic state. Rather the group differences are distributed across four dynamic states (states 1, 2, 3, and 4). This distributive nature of the group differences could be one reason they were not detected in the static FNC, since that metric only shows the average FNC for the run. Also the dynamic states in **Figure 4** show higher *p*-values for several *t*-tests between ICN components for different groups, which did not pass multiple comparison correction tests. With a larger sample size, more significant group difference could be revealed.

Significant between-group differences in connectivity strength were found in several intrinsic networks including sub-cortical, VIS, auditory, SM, CC, DM and cerebellum networks. Several components in the default-mode network (DMN) including DMPFC, right and left angular gyri (AG), and right and left precuneus showed significant connectivity differences with the components in VIS, CC, SM, auditory, and CB networks. Previous studies suggest that DMN may distinguish SZ and bipolar patients from HCs (Zhou et al., 2007, 2008; Calhoun et al., 2008b, 2011). The majority of previous studies report reduced task-related suppression in the DMN in SZ (Zhou et al., 2007, 2008; Jafri et al., 2008; Bluhm et al., 2009; Jann et al., 2009; Kim et al., 2009; Park et al., 2009; Pomarol-Clotet et al., 2010; Wang et al., 2011). Studies showed that failure to deactivate default-mode regions corresponded to gray matter losses in the dorsal ACC and medial prefrontal cortex regions (Zhou et al., 2008; Pomarol-Clotet et al., 2010; Skudlarski et al., 2010; Salgado-Pineda et al., 2011). However, as mentioned earlier, both increased and decreased FC have been reported in the DMN in SZ. Medial prefrontal cortex is a region known to be associated with information processing when more than one course of action may be required, such as representing the thoughts, actions, and feelings of others across time (Gilbert et al., 2006). Several studies of both SZ and BP (Öngür et al., 2010; Meda et al., 2012; Khadka et al., 2013) have reported subgenual and medial prefrontal abnormalities in bipolar patients and dorsal medial prefrontal abnormalities in SZ patients. (Huang et al., 2010) reported decreased amplitude of low frequency fluctuation (ALFF) in the medial prefrontal regions in never treated SZ patients, and found to become normalized with antipsychotic therapy (Sambataro et al., 2009; Lui et al., 2010).

Another DMN component found in our analysis is the angular gyrus (AG), which is known to be involved in language processing (Hall et al., 2005; Binder et al., 2009; Price, 2010; Clos et al., 2014), as well as memory and social cognition. Therefore, AG dysregulation can help differentiate SZ and bipolar patients from HCs. Our study showed greater connectivity in SZ between the component pair STG and left AG. Notably, several studies also found FC abnormalities in STG, which is a major part of the dominant hemisphere language network. Also both structural and functional abnormalities in the STG have been demonstrated in SZ patients in multiple studies as well as in psychotic BP and constitute the best-replicated brain differences correlating with the severity of psychotic symptoms in SZ, most specifically auditory hallucinations and formal thought disorder collectively; abnormalities in these regions likely underpin psychotic phenomena (Aguayo, 1990; Swerdlow, 2010; Fusar-Poli et al., 2011). In our study, group variations in connectivity strength were observed in several temporal lobe components [STG, bi-fusiform gyrus (FFG) and left inferior temporal gyrus (ITG)], known to process auditory information (Kim et al., 2009; Sui et al., 2011). This reinforces the fact that aberrant temporal lobe coherence patterns may exhibit significant abnormality in both SZ, and to a lesser extent BP (Pearlson, 1997; Calhoun et al., 2008b). These findings may be useful in explaining the language and thought disruptions in SZ.

Our study showed two other DMN components, left and right precuneus, which are involved in a wide spectrum of highly integrated tasks, including episodic memory (Cabeza and Nyberg, 2000; Rugg and Henson, 2002), mental imagery recall (Shallice et al., 1994; Fletcher et al., 1996), and self-processing operations, such as first-person perspective taking (Cavanna and Trimble, 2006). Garrity et al. (2007), higher positive symptoms were correlated with increased deactivation in the medial frontal gyrus, precuneus and the left middle temporal gyrus (MiTG). Compared to SZ patients, HCs showed greater connectivity between left cerebellum and both left and right precuneus. The cerebellum may influence motor systems by estimating inconsistencies between intention and action and by adjusting the motor operations appropriately (Kandel et al., 2000), as well playing a role in cognition and emotion (Schmahmann and Caplan, 2006). Prior studies reveal impaired functional integration of cerebellum in SZ (Honey et al., 2005; Becerril et al., 2011). Collin et al. (2011) proclaimed the FC to other brain regions [left thalamus, middle cingulate gyrus, and supplementary motor area (SMA)] to be disconnected from the cerebellum in SZ patients.

In our study, several SM components including SMA, right and left motor, VM, supramarginal gyrus (SmG) and paracentral showed between-group connectivity differences that were distributed across different dynamic states. (Jeong et al., 2009) reported decreased correlation of the left inferior frontal gyrus (IFG) with left middle temporal gyrus (MTG)/ left superior temporal sulcus, left SPL/supramarginal gyrus and other brain regions. Our results showed connectivity differences between SmG and other brain components [with lingual gyrus (LG) in HC < SZ; with VM in SZ > BP; with bi-FFG in HC < BP]. Previous studies found impaired FC between cerebellum and LG in SZ patients (Collin et al., 2011).

Other findings in our analysis include connectivity differences in several CC components [left ITG, left middle frontal gyrus (MiFG), MTG, left IFG, left superior medial gyrus (SMG), and SPL] with components from other brain networks. Abnormal FC in left IFG, MiFG, and IFG was found in SZ patients (Jeong et al., 2009; Müller et al., 2013).

Another key finding in our study is greater connectivity in HCs between putamen tail and VM regions, compared to SZ patients. The putamen may be involved in the generation of spontaneous language, and linked to auditory/verbal hallucinations (Hoffman and Hampson, 2012). Several SZ studies showed FC anomalies in the putamen (Hoffman et al., 2011; Hoffman and Hampson, 2012; Tu et al., 2012).

## LIMITATIONS AND FUTURE DIRECTIONS

Several experimental and methodological limitations must be considered while performing the sliding-window analysis method and interpreting results. One limitation is that the non-stationary noise sources in fMRI time series can influence changes in FC over time. Synchronous global modulations of fMRI time series can be caused by variations in respiratory and cardiac rates, as they predominantly occupy the low frequencies (<0.1 Hz; Wise et al., 2004; Chang and Glover, 2009). Also head motion could generate spatially structured artifacts in FC (Power et al., 2012; Yan et al., 2013). Even though ICA reasonably separates the component sources for sliding-window analysis, it may not have completely separated the effects from other sources of interest. Therefore, to interpret the dynamic results, efficient denoising as well as recording of respiration and cardiac events should be considered. In the current study we performed careful quality control as well as incorporating multiple motion regression steps to mitigate against the impact of motion.

Another important issue for sliding-window analysis is the choice of window size. (Sakoǧlu et al., 2010) reported that the ideal window size should be able to estimate FC variability and capture the lowest frequencies of interest in the signal, as well as to detect interesting short-term effects. In this study, dynamics were estimated using an empirically validated fixed sliding-window of 22TRs (33s) similar to that used in (Allen et al., 2012). Future work should evaluate changes across a variety of windows lengths that could be performed using separate windows (Cribben et al., 2012) or perhaps combined with multi-scale approaches such as wavelet transform (Chang and Glover, 2010).

Several recent studies on microstate-based EEG-fMRI resting-state datasets have showed that EEG microstates and some number of fMRI-based ICNs show correspondence between themselves (Britz et al., 2010; Musso et al., 2010; Yuan et al., 2012). A brain microstate can be defined as a functional/physiological state during which specific neural processes occur (Musso et al., 2010). Using concurrent EEG-fMRI data, the underlying physiological correlates of these dynamic states can be well assessed as demonstrated in (Allen et al., 2013).

We characterized FC as the covariance between ICN timecourses. Characterization of FC matrices based on higher-order statistics (e.g., mutual information) or lag-insensitive measures (e.g., cross-correlation) could efficiently recover the underlying biological structure of networks. Another limitation of the study is that smaller acquisition parameters may not lead to optimum results by exploring all possible aspects of dynamic changes in FC. Each subject in this study was scanned for only 5 min, which is probably not optimal for considering the rate of change in dynamic states. A longer acquisition time (∼10 min) is recommended for a more accurate estimation of connectivity dynamics. To identify centroids of dynamic FC we used k-means clustering, which has several limitations, including difficulty in separating clusters with different sizes and densities, and a high susceptibility to outliers. Future work could include application of alternative clustering models (fuzzy-clustering or density-based clustering techniques) in the connectivity dynamics. Future work focusing on an improved understanding of the association between disease and connectivity dynamics could actually enrich our knowledge of the dynamic properties of the healthy functional brain. In addition, recent work has shown that there are time-varying changes not only in the covariance but also in the associated spatial patterns (Ma et al., 2014). Future studies to characterize both covariance and spatial changes over time are warranted.

## CONCLUSION

We have performed, to our knowledge, the first whole-brain characterization of intrinsic regional differences in FC variability and a comprehensive analysis of discrete FC states in SZ, BP and HCs. One key finding was the aberrant FC patterns found in several default-mode components including DMPFC, bilateral angular gyrus, and bilateral precuneus, in the patient groups. Other significant findings include connectivity anomalies in VIS, SM and cognitive control networks in both patient groups. These findings could be used as distinctive characteristic markers in SZ and BP, and also could help diagnose the patients based on their biological features, rather than exclusively depending on cross-sectional clinical symptoms and information on longitudinal course and outcome.

## Conflict of Interest Statement

The authors declare that the research was conducted in the absence of any commercial or financial relationships that could be construed as a potential conflict of interest.

## Footnotes

1 http://www.fil.ion.ucl.ac.uk/spm/software/spm5

2 http://www.mni.mcgill.ca/
