# Supplementary material for: Dynamic connectivity states estimated from resting fMRI Identify differences among Schizophrenia, bipolar disorder, and healthy control subjects
Source: Front Hum Neurosci. 2014 Nov 7;8:897. doi: 10.3389/fnhum.2014.00897 (PMC4224100; doi:10.3389/fnhum.2014.00897)
Supplement: Supplementary file 1 [file Table_1.DOCX]

***Supplementary Material***

**Dynamic Connectivity States Estimated from Resting fMRI Identify Differences among Schizophrenia, Bipolar Disorder, and Healthy Control Subjects**

**Barnaly Rashid_1_^1,2^, Eswar Damaraju_2_^1,2^, Godfrey D Pearlson_3_^3,4,5^, Vince D. Calhoun_4_^1,2,3,4 *^**

^1^ The Mind Research Network, Albuquerque, New Mexico, USA

^2^ Department of Electrical and Computer Engineering, University of New Mexico, Albuquerque, New Mexico, USA

^3^ Olin Neuropsychiatry Research Center, Institute of Living, Hartford, Connecticut, USA

^4^ Departments of Psychiatry and ^5^ Neurobiology, Yale University School of Medicine, New Haven, Connecticut, USA

*** Correspondence:** Vince D. Calhoun, The Mind Research Network, 1101 Yale Blvd NE, Albuquerque, NM 87106, USA

e-mail: vcalhoun@mrn.org

1. **Supplementary Table**

**Supplementary Table 1. Peak Coordinates of ICNs**

| ICN regions | BA | t_max_ | Peak (mm)  X Y Z |
| --- | --- | --- | --- |
| **Subcortical Networks** | | | |
| Amygdala (48)  Left Amygdala  Right Hippocampus | 34  34 | 42.95  44.01 | [-24 -6 -18]  [27 -6 -18] |
| Thalamus (61)  L Thalamus  Insula Lobe | 13 | 64.37  17.28 | -12 -18 3  -36 -6 0 |
| Putamen (78)  R Putamen  L Putamen |  | 63.69  58.30 | 30 0 3  -27 -6 9 |
| Putamen (91)  L Putamen | 34 | 73.94 | -24 9 -6 |
| **Auditory Networks** | | | |
| STG (36)  L Superior Temporal Gyrus  R Superior Temporal Gyrus | 41  13 | 50.57  39.86 | -48 -30 12  45 -15 3 |
| **Visual networks** | | | |
| FFG (31)  L Fusiform Gyrus  R Fusiform Gyrus | 37  37 | 47.14  34.56 | -36 -57 -15  36 -48 -21 |
| Lingual Gyrus (10)  L Lingual Gyrus | 18 | 58.58 | -12 -84 -12 |
| Inferior Occipital Gyrus (11)  R Inferior Occipital Gyrus  L Middle Occipital Gyrus | 18  18 | 54.54  54.21 | -27 -99 -3  27 -99 -6 |
| Cuneus (16)  L Cuneus | 18 | 64.24 | 0 -81 24 |
| Calcarine (21)  L Calcarine | 17 | 58.26 | 6 -93 3 |
| Calcarine (29)  R Calcarine | 30 | 62.36 | 12 -69 9 |
| Inferior Occipital Gyrus (32)  L Inferior Occipital Gyrus  R Fusiform Gyrus | 19  18 | 44.72  17.32 | -36 -75 -3  30 -78 0 |
| Lingual Gyrus (33)  R Lingual Gyrus | 19 | 51.03 | 21 -54 -9 |
| Middle Occipital Gyrus (54)  L Middle Occipital Gyrus  R Middle Occipital Gyrus | 19  19 | 47.16  47.35 | -33 -90 12  30 -93 12 |
| Middle Occipital Gyrus (87)  R Middle Occipital Gyrus  L Middle Occipital Gyrus | 19  19 | 50.78  45.91 | 33 -78 33  -30 -78 36 |
| **Sensorimotor Networks** | | | |
| Postcentral (1)  R Postcentral Gyrus  L Postcentral Gyrus | 4  6 | 57.37  54.17 | 60 -3 24  -54 -9 30 |
| Paracentral (9)  L Paracentral Lobule | 6 | 65.05 | 0 -24 63 |
| Postcentral (14)  L Postcentral Gyrus | 4 | 51.81 | -36 -24 51 |
| Precentral (15)  R Precentral Gyrus  L Cerebellum (VI) | 3 | 47.23  16.27 | 39 -21 54  -21 -51 -24 |
| Superior Medial Gyrus (27)  L Superior Medial Gyrus | 8 | 43.82 | 3 30 54 |
| SMA (35)  R supplementary motor area | 24 | 43.01 | 12 -6 51 |
| SupraMarginal (38)  L SupraMarginal Gyrus  R Postcentral Gyrus  L Inferior Frontal Gyrus  R Inferior Frontal Gyrus | 3  3  44  9 | 45.53  40.27  21.43  15.86 | -60 -21 36  57 -18 33  -54 9 24  60 12 27 |
| Middle Cingulate Cortex (80)  L Middle Cingulate Cortex  R Precentral Gyrus  L Postcentral Gyrus | 24  6  6 | 50.35  18.61  17.51 | -3 -18 39  42 -15 36  -42 -15 36 |
| SMA (70)  L SMA  R Precentral Gyrus  L Postcentral Gyrus  L Temporal Pole  R Temporal Pole | 6  6  4  22  38 | 50.76  19.80  17.14  17.33  17.39 | 0 9 51  51 -6 45  -48 -9 51  -54 12 -9  54 15 -9 |
| **Cognitive Control** | | | |
| Inferior Temporal Gyrus (64)  L Inferior Temporal Gyrus  R Inferior Temporal Gyrus | 37  37 | 47.57  20.76 | -45 -54 -9  54 -48 -9 |
| Inferior Temporal Gyrus (66)  R Inferior Temporal Gyrus  L Inferior Occipital Gyrus | 37  19 | 46.77  22.17 | 45 -66 -6  -48 -75 -6 |
| Middle Temporal Gyrus (92)  R Middle Temporal Gyrus  L Middle Temporal Gyrus | 21  21 | 51.20  41.31 | 57 -21 -9  -60 -30 -3 |
| Middle Temporal Gyrus (42)  R Middle Temporal Gyrus  L Middle Temporal Gyrus | 22  39 | 42.62  42.91 | 51 -60 15  -51 -63 15 |
| Superior Parietal Lobule (60)  R Superior Parietal Lobule | 7 | 48.93 | 15 -51 63 |
| Inferior Parietal Lobule (63)  L Inferior Parietal Lobule  R SupraMarginal Gyrus  R Inferior Frontal Gyrus | 40  40  47 | 47.00  47.42  16.04 | -57 -42 36  57 -45 33  51 18 -6 |
| Superior Parietal Lobule (94)  R Superior Parietal Lobule | 7 | 52.33 | 27 -63 57 |
| Inferior Frontal Gyrus (95)  L Inferior Frontal Gyrus(p. Triangularis)  L Inferior Parietal Lobule  L Inferior Temporal Gyrus | 46  40  20 | 34.68  28.31  25.66 | -45 33 15  -54 -42 48  -54 -51 -15 |
| Inferior Frontal Gyrus (57)  R Inferior Frontal Gyrus  L Inferior Frontal Gyrus  Right Inferior Parietal Lobule | 9  46  40 | 50.30  30.38  16.96 | 45 12 30  -45 18 27  36 -51 51 |
| Middle Frontal Gyrus (84)  L Middle Frontal Gyrus | 10 | 45.48 | -27 48 21 |
| Inferior Frontal Gyrus (86)  L Inferior Frontal Gyrus  L Supplementary motor area  L Middle Temporal Gyrus | 47  6  21 | 44.97  28.30  23.46 | -48 24 -3  -3 18 60  -60 -36 -3 |
| Superior Medial Gyrus (88)  L Superior Medial Gyrus  R Insula Lobe  L Insula Lobe | 9  47  47 | 45.19  31.73  25.87 | 3 36 33  33 21 -9  -33 18 -9 |
| Insula Lobe  R Insula Lobe  L Insula Lobe | 47  47 | 58.98  48.66 | 42 12 -3  -39 18 -3 |
| Middle Frontal Gyrus (68)  L Middle Frontal Gyrus | 10 | 43.44 | -33 57 3 |
| **Default Mode Networks** | | | |
| Superior Medial Gyrus (46)  L Superior Medial Gyrus  R Inferior Frontal Gyrus | 9  47 | 60.75  16.89 | 0 54 24  48 36 -9 |
| Precuneus (25)  R Precuneus | 7 | 68.05 | 3 -51 45 |
| Anterior Cingulate Cortex (43)  L Anterior Cingulate Cortex | 32 | 63.24 | -3 42 3 |
| Angular Gyrus (65)  L Angular Gyrus  L Precuneus  L Middle Frontal Gyrus  L Middle Temporal Gyrus  R Inferior Parietal Lobule | 40  7  8  21  40 | 56.15  26.66  24.77  19.47  19.12 | -48 -63 39  -3 -60 33  -45 18 45  -63 -42 -9  51 -57 45 |
| Angular Gyrus (37)  R Angular Gyrus  L Angular Gyrus  R Middle Frontal Gyrus  R Middle Cingulate Cortex | 39  39  8  31 | 65.56  23.19  17.43  17.67 | 51 -63 36  -39 -69 39  36 24 54  6 -39 36 |
| Precuneus (69)  L Precuneus | 31 | 68.27 | 0 -60 27 |
| Precuneus (72)  L Precuneus  R Middle Cingulate Cortex | 7  23 | 62.53  29.56 | -6 -72 39  0 -21 30 |
| Middle Cingulate Cortex (75)  R Middle Cingulate Cortex | 31 | 59.83 | 6 -33 33 |
| **Cerebellar Networks** | | | |
| Cerebellum (3)  L Cerebellum (VIII)  R Cerebellum (Crus 2) |  | 56.24  21.93 | -36 -54 -45  42 -54 -39 |
| Cerebellum (30)  L Cerebellum (VI) |  | 56.64 | -27 -63 -27 |
| Cerebellum (59)  R Cerebellum (Crus 2)  R Cerebellum (IX) | 18 | 52.08  19.18 | 21 -87 -33  3 -48 -36 |
